# Primary care focus and utilization in the Medicare shared savings program accountable care organizations

**DOI:** 10.1186/s12913-017-2092-8

**Published:** 2017-02-15

**Authors:** Lindsey A. Herrel, John Z. Ayanian, Scott R. Hawken, David C. Miller

**Affiliations:** 1 Dow Division of Health Services Research, University of Michigan, Ann Arbor, Michigan USA; 2 Department of Urology, University of Michigan, Ann Arbor, Michigan USA; 3 Institute for Healthcare Policy and Innovation, University of Michigan, Ann Arbor, Michigan USA; 4 Division of General Medicine, Medical School, University of Michigan, Ann Arbor, Michigan USA; 5 Department of Health Management and Policy, School of Public Health, University of Michigan, Ann Arbor, Michigan USA; 6 Gerald R. Ford School of Public Policy, University of Michigan, Ann Arbor, Michigan USA

**Keywords:** Accountable care organizations, Primary care, Utilization

## Abstract

**Background:**

Although Accountable Care Organizations (ACOs) are defined by the provision of primary care services, the relationship between the intensity of primary care and population-level utilization and costs of health care services has not been examined during early implementation of Medicare Shared Savings Program (MSSP) ACOs. Our objective was to evaluate the association between primary care focus and healthcare utilization and spending in the first performance period of the Medicare Shared Savings Program (MSSP) Accountable Care Organizations (ACOs).

**Methods:**

In this retrospective cohort study, we divided the 220 MSSP ACOs into quartiles of primary care focus based on the percentage of all ambulatory evaluation and management services delivered by a PCP (internist, family physician, or geriatrician).

Using multivariable regression, we evaluated rates of utilization and spending during the initial performance period, adjusting for the percentage of non-white patients, region, number of months enrolled in the MSSP, number of beneficiary person years, percentage of dual eligible beneficiaries and percentage of beneficiaries over the age of 74.

**Results:**

The proportion of ambulatory evaluation and management services delivered by a PCP ranged from <38% (lowest quartile, ACOs with least PCP focus) to >46% (highest quartile, ACOs with greatest PCP focus). ACOs in the highest quartile of PCP focus had higher adjusted rates of utilization of acute care hospital admissions (328 per 1000 person years vs 292 per 1000 person years, *p* = 0.01) and emergency department visits (756 vs 680 per 1000 person years, *p* = 0.02) compared with ACOs in the lowest quartile of PCP focus. ACOs in the highest quartile of PCP focus achieved no greater savings per beneficiary relative to their spending benchmarks ($142 above benchmark vs $87 below benchmark, *p* = 0.13).

**Conclusions:**

Primary care focus was not associated with increased savings or lower utilization of healthcare during the initial implementation of MSSP ACOs.

## Background

The Affordable Care Act (ACA) granted the Centers for Medicare and Medicaid Services (CMS) the authority to establish Medicare Shared Savings Program (MSSP) Accountable Care Organizations (ACOs) [1]. The risk-bearing payment systems accepted by MSSP ACOs are designed to enhance accountability and care coordination among groups of providers. Accordingly, this program has grown rapidly to include 405 ACOs caring for approximately 7.2 million Medicare beneficiaries as of January 2015 [2].

A primary requirement for participation in the MSSP is that an ACO provides primary care services for at least 5000 Medicare beneficiaries. Consequently, these new organizations differ widely with respect to both physician composition and the distribution of care provided by primary care physicians (PCPs) and specialist physicians. It is unknown, however, whether such differences influence ACO performance. Evaluation of the Pioneer ACO program, a predecessor to the MSSP, noted smaller increases in Medicare expenditures coupled with decreased utilization of primary care visits, procedures, imaging and testing compared to non-ACOs [3]. Specialists are often gatekeepers to high cost services including procedures and imaging studies, and therefore may play an important role in generating savings if they are engaged in an ACO. ACOs also vary in their leadership (physician versus hospital leads), location (rural versus urban) and size, all of which can influence the physician composition and patient populations served by the ACO. While some believe that the optimal ACO model involves provision of ambulatory care mainly by PCPs, [4–6] the relationship between primary care focus and utilization and costs of health care services has not been examined during early implementation of MSSP ACOs.

To address this gap, we used data from CMS to measure the PCP focus of MSSP ACOs based on the percentage of evaluation and management services provided by primary care physicians. We then compared utilization of health care services and savings over benchmark during the first performance period for MSSP ACOs according to their level of PCP focus.

## Methods

### Data source

We used the CMS Shared Savings Program public-use file [7] released in January 2015 to perform these analyses. This file provides ACO-level data from the first performance period (ending December 2013) for the 220 MSSP ACOs that enrolled from April 2012 through January 2013. Because we analyzed organizational data from ACOs and not individual-level data, our study was deemed not regulated by the University of Michigan Institutional Review Board.

The available data include summary information on ACO characteristics, as well as measures of benchmark spending, and health services utilization and expenditures during the performance period. In terms of benchmark spending, the CMS Office of the Actuary calculates this metric for each MSSP ACO based on the three years of spending (under Medicare Fee-For-Service Parts A and B) prior to the performance period for attributed beneficiaries, with the most recent year weighted most heavily. The benchmark estimates are risk adjusted using the CMS Hierarchical Condition Categories (HCC), and the national growth rate in Medicare spending is applied to obtain the final benchmark spending [8]. Demographic scores (recalculated annually for all ACO beneficiaries) and CMS-HCC risk scores (calculated for new ACO enrollees only) are combined to provide a case mix adjustment that is updated annually based on the current roster of assigned ACO beneficiaries.

### Measurement and classification of PCP focus

Consistent with the statutory definition in the ACA, ambulatory evaluation and management services are defined by Healthcare Common Procedure Coding System codes 99201-99215, 99304-99350, G0402, G0438, G0439, and by revenue center codes 0521, 0522, 0524, 0525 when submitted by a federally qualified health center or rural health clinic. Medicare beneficiaries are assigned to an ACO when the plurality of their primary care services are provided by a physician who aligns with an ACO via a tax identification number. Once the beneficiary is assigned, all Medicare services and expenditures related to their care are attributed to the ACO whether this care occurs within the ACO or outside the ACO. Currently, expenditures for MSSP ACOs are calculated based on Medicare spending only and not Medicaid or private insurer payments.

We based our measure of primary care focus on the percentage of such services for ACO beneficiaries that were delivered by any primary care physician, including internists, family medicine physicians, geriatricians, and pediatricians, during the first performance period. We calculated this measure for each ACO by dividing the number of evaluation and management visits provided by a PCP per 1000 person years by the total number of evaluation and management visits per 1000 person years. Both of these variables were provided in the SSP files. Using this measure, we sorted the MSSP ACOs into quartiles of PCP focus based on their percentage of evaluation and management services delivered by primary care physicians.

### Outcome measures

From the SSP files, we also identified several measures related to utilization of health care services, including the number of acute care hospital discharges per 1000 person years, and the number of emergency department visits per 1000 person years. Several summary measures of ACO spending were also available, including benchmark (i.e., pre-ACO implementation) and performance period expenditures.

For analytic purposes, we first annualized the expenditure metrics to account for variability in ACO start dates. Next, we divided the annualized measures of spending by the number of assigned beneficiary person years (i.e., number of beneficiaries standardized for the length of time they are attributed to the ACO) to calculate the annual spending per beneficiary for each MSSP ACO. Finally, we measured savings per beneficiary for each ACO by subtracting the annualized per beneficiary expenditures for the performance period from the annualized per beneficiary benchmark spending. For this measure, positive and negative values indicate cost savings and losses, respectively.

### Statistical analysis

We used Student’s *t*-test and ANOVA to compare characteristics of ACOs with the least and greatest PCP focus. We then used zip codes provided by CMS and ArcGIS software version 10 (Esri, Redlands, California) to map the location of ACOs falling in the highest and lowest quartiles of PCP focus.

We fit multivariable linear models to estimate the adjusted association of PCP focus with ACO-level metrics of utilization and spending, controlling for the percentage of non-white patients, percentage of dual eligible beneficiaries, percentage of beneficiaries over 74 years old, geographic region by census division (New England, Middle Atlantic, East North Central, West North Central, South Atlantic, East South Central, West South Central, Mountain, Pacific), rurality, number of months enrolled in the MSSP, and number of beneficiary person years. We selected the covariates for our model *a priori* based on hypotheses and informed by prior work suggesting that these factors may be associated with utilization and spending [9, 10]. For example, older age, non-white race and dually eligible beneficiaries have been associated with higher health care expenditures. From these models, we estimated adjusted measures of utilization and spending for each ACO and compared these across strata of PCP focus. Utilization metrics included number of E&M visits, acute care hospital discharges, readmissions (30 days), post-hospitalization visits (30 days), emergency department visits and discharges to a skilled nursing facility. Spending metrics included physician spending, acute care hospital spending, skilled nursing facility spending and annual per beneficiary savings. Finally, we also evaluated total expenditures.

We performed three additional sensitivity analyses. First, to determine if our findings were robust to the use of quartiles, we performed a linear regression to evaluate utilization outcomes using the proportion of E&M services provided by a PCP (continuous variable) as our dependent variable. Second, we performed the same analyses listed above using terciles rather than quartiles. Finally, we used a log-log model to evaluate our spending metrics with the proportion of E&M services provided by a PCP as a continuous dependent variable. *P* values <0.05 were considered statistically significant. All statistical analyses were performed with Stata version 13 (StataCorp LP, College Station, Texas).

## Results

We identified 220 ACOs that joined the MSSP from April 2012 through January 2013. Overall, these 220 MSSP ACOs had total benchmark spending set at $42.5 billion and total expenditures of $42.3 billion for the more than 3 million beneficiaries cared for during the first performance period, resulting in more than $230 million in estimated savings.

**Fig. 1:**
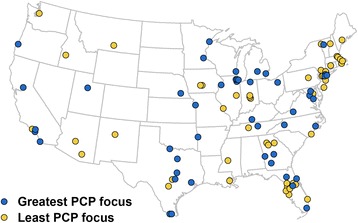
Geographic distribution of ACOs with the least and greatest PCP focus (*p* = 0.02).* (*2 ACOs in Puerto Rico are not shown; both were in the group with greatest PCP focus). Source: Created using ArcGIS software. Permission granted for reproduction

We classified ACOs into four equal quartiles of PCP focus defined by the following proportions of evaluation and management services delivered by a PCP: 3.3–38.1% (lowest quartile, referred to throughout the manuscript as least PCP focus), 38.1–42.0% (quartile 2), 42.0–46.4% (quartile 3), and 46.5–64.8% (highest quartile, referred to as greatest PCP focus). As illustrated in Fig. 1, there were significant differences in the geographic distribution of ACOs in the highest and lowest quartiles of PCP focus during 2012 and 2013; ACOs with the greatest degree of PCP focus were more common in the Midwest, while those with the least PCP focus were more common in the Northeast (*p* = 0.02).

**Fig. 2:**
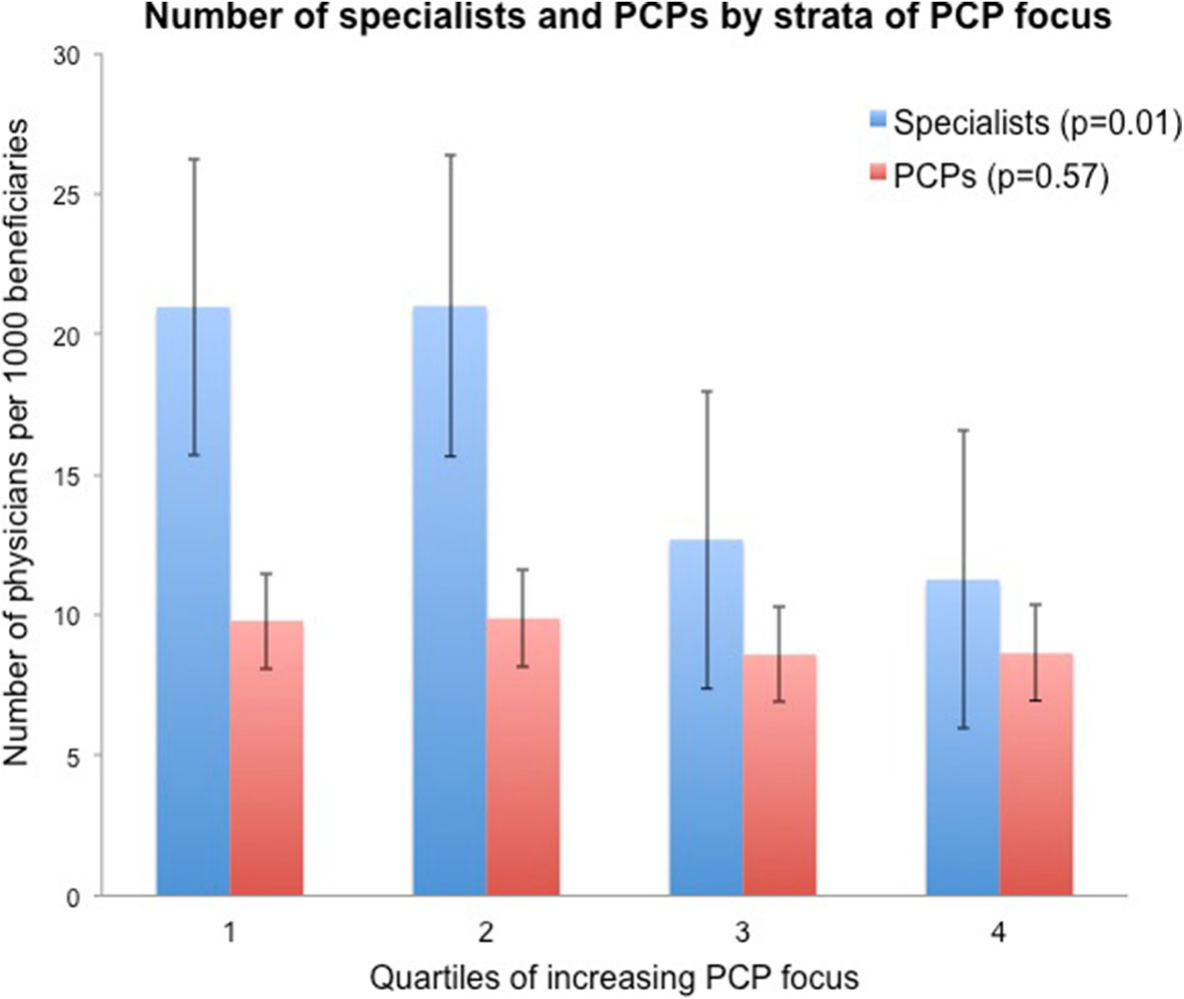
Mean number of specialists and PCPs in MSSP Accountable Care Organizations according to strata of primary care focus

Table 1 compares characteristics of ACOs with the greatest and least PCP focus and reveals a similar composition of beneficiaries (including overall number, as well as those with end stage renal disease and those on disability) with the exception that ACOs with the greatest PCP focus have a higher proportion of non-white and dual-eligible beneficiaries. Whereas the numbers of PCPs per 1000 beneficiaries did not differ significantly across quartiles (*p* = 0.57), the number of participating specialists was almost twice as large in the two lowest quartiles of PCP focus compared with the two highest quartiles (*p* = 0.01) (Fig. 2).

**Table 1:** Characteristics of ACOs with least and greatest PCP focus

Mean (SD)	Least PCP focus	Greatest PCP focus	p-value
Number assigned beneficiaries	18,504 (16,137)	14,751 (19,179)	0.27
Mean length of performance period (months)	15.5 (3.5)	15.6 (3.6)	0.94
Percentage of minority beneficiaries	13.8 (13.7)	24.5 (23.5)	0.004
Mean percentage of ESRD patients	1.01 (0.7)	1.26 (0.8)	0.09
Mean percentage of disabled patients	15.2 (8.8)	15.7 (6.2)	0.73
Mean percentage of dual-eligible beneficiaries	6.3 (5.9)	14.1 (18.7)	0.004
Rural (%)	3.6	7.3	0.40
Census Division (%)			0.01
New England	18.2	1.8	
Middle Atlantic	20.0	10.9	
East North Central	7.3	20.0	
West North Central	5.5	9.1	
South Atlantic	23.6	23.6	
East South Central	5.5	3.6	
West South Central	7.3	14.6	
Mountain	9.1	1.8	
Pacific	3.6	9.1	
Puerto Rico	0.0	5.5	

*ESRD* End-stage renal disease

Table 2 presents measures of utilization and expenditures for ACOs in the highest compared with lowest quartiles of PCP focus. ACOs with the greatest PCP focus had more total E&M visits, including a comparatively higher number of PCP visits and a lower number of specialist visits. During the first performance period, MSSP ACOs with the greatest PCP focus had higher adjusted rates of acute care hospital admissions (328 per 1000 person years vs 292 per 1000 person years, *p* = 0.01) and emergency department visits (756 vs 680 per 1000 person years, *p* = 0.02) compared with ACOs with the least PCP focus. No significant difference was evident in mean savings per beneficiary relative to benchmark spending levels across quartiles of PCP focus. Additionally, we noted no differences in total expenditures with $10,068 per beneficiary per year for low PCP focus ACOs and $10,723 for ACOs with the greatest PCP focus, *p* = 0.15.

**Table 2:** Utilization and spending in ACOs with least and greatest PCP focus

Metric (95% CI)	Least PCP focus	Greatest PCP focus	*p*-value
Total E&M visits per 1000 person years	9957 (9511–10,403)	10,664 (10,139–11,188)	0.04
E&M visits by a PCP per 1000 person years	3131 (2820–3440)	5561 (5202–5920)	<0.001
E&M visits by a specialist per 1000 person years	5065 (4617–5513)	4319 (4044–4595)	0.005
Acute care hospital discharges per 1000 person years^a^	292 (274–311)	328 (309–348)	0.01
30-day acute care readmissions per 1000 discharges^a^	146 (141–152)	156 (150–162)	0.02
Post discharge (30 day) provider visits per 1000 discharges^a^	757 (748–765)	776 (767–785)	0.01
Skilled nursing facility discharges per 1000 person years^a^	73 (61–85)	106 (93–119)	0.001
Emergency Department visits per 1000 person years^a^	680 (639–722)	756 (711–800)	0.02
Physician/supplier spending per assigned beneficiary^a^	$3296 (3112–3479)	$3165 (2970–3360)	0.36
Acute care hospital spending per assigned beneficiary^a^	$2774 (2561–2987)	$3180 (2953–3407)	0.02
Skilled nursing facility spending per assigned beneficiary^a^	$818 (642–993)	$1199 (1063–1437)	0.002
Savings per beneficiary per year	$87 ($-104–$278)	$-142 ($-346–$61)	0.13

^a^Adjusted for number of beneficiaries, percent non-white beneficiaries, percent dual eligible, percent age over 74 years, census division and months in ACO

*E&M* Evaluation and management

*PCP* Primary care physician

Our sensitivity analyses revealed no substantive changes from our primary findings. First, using the proportion of E&M visits by a PCP as a continuous variable, our findings of significantly higher rates of utilization remained for skilled nursing facility and hospital admissions, as well as readmissions and post discharge provider visits (all *p*-values <0.05). When we divided ACOs into terciles of PCP focus we demonstrated higher rates of utilization of post discharge provider visits, skilled nursing facility discharges and emergency department visits and no differences in savings for ACOs in the highest tercile of PCP focus. Using a log-log model to evaluate our spending outcomes, we similarly demonstrated no difference in total expenditures, benchmark spending or total savings (all *p* > 0.05).

## Discussion

MSSP ACOs differ significantly with respect to primary care focus, as measured by the percentage of E&M services provided by primary care physicians. Notably, in the first performance period, ACOs with the greatest PCP focus utilized more hospital care, suggesting that—during the earliest phases of ACO implementation—primary care intensity is not clearly associated with lower utilization. Moreover, ACOs with the greatest degree of PCP focus achieved no more savings than their less PCP focused counterparts.

Our findings of increased utilization and no difference in savings for ACOs with a greater degree of PCP focus add to a growing body of literature examining factors that may influence patterns of healthcare use and savings in these organizations. While these results may appear counter to prior work indicating that increasing primary care focus may improve access, quality and cost; [11] this relationship likely depends on both contextual (e.g., ACO size) [5] and patient factors (e.g., comorbidities) [12] that vary across MSSP organizations. For example, ACOs in more rural locations or those with a smaller physician panel may have fewer specialist physicians to manage complex medical conditions (e.g, CHF managed by a cardiologist versus a PCP). ACOs in these rural areas may face challenges with both specialty and primary care physician shortages. Similarly, whether hospital- or physician-led, ACO leadership will be incentivized differently and will need to adapt and respond to their particular patient population and case-mix as improvements in population health are rewarded [13]. ACOs that have independent ownership have demonstrated greater savings than hospital led organizations early in the MSSP [14]. Additionally, location and prior spending plays a role as ACOs in higher spending regions have been shown to yield greater savings during the performance period, perhaps from addressing the “lowest hanging fruit” of cost savings [15]. Taken together, our results add to current literature that suggests a complex relationship between individual organizational attributes (e.g., degree of integration, geography, ACO size, patient case-mix) and healthcare spending that will impact how the structure and composition of ACOs evolve over time.

Our study has several limitations. First, because the Shared Savings Program public-use file provides summarized information at the ACO level, our findings are subject to the ecological fallacy. In other words, although greater PCP focus was associated with higher spending when aggregated to the ACO level, this may not be the case for individual physicians or beneficiaries. Nonetheless, our methods of evaluation (i.e., ACO-level) are consistent with the approach used by CMS for measuring quality and determining shared savings or losses in the MSSP program. Second, because the SSP dataset does not include beneficiary-level information, we cannot fully account for differences in patient complexity across ACOs. However, our multivariable models did adjust for measurable ACO characteristics that may influence utilization and spending, including geographic region, rurality, proportion of non-white patients and those with dual-eligible status. In addition, our results compare utilization and savings from the first performance period, and these findings may shift over time as ACOs refine their ability to improve quality and reduce costs. Finally, this study only included MSSP ACOs and therefore our results may not be generalizable to other ACOs, including the Pioneer ACO that have demonstrated modest savings in their early implementation [3, 16].

Our measurement of PCP focus also has limitations. First, this utilization-based metric does not capture quality, care coordination, or other aspects of care delivery that may have important implications for utilization and spending at the ACO level. Additionally, because we distinguish between specialist versus primary care oriented advanced practice providers we elected to not include these services. Second, the thresholds for our PCP focus variable were selected to ensure an equal number of ACOs in each quartile. As such, they do not necessarily represent clinically meaningful thresholds in the provision of primary care services. Third, E&M services provided in patient homes or nursing homes are contained within the PCP metric. These beneficiaries may be responsible for a larger number of visits and are likely to be sicker and incur greater healthcare costs, which may contribute to differences in utilization between ACOs with the least versus greatest PCP focus. Finally, our measurement of PCP focus may be a surrogate for other organizational attributes that influence utilization and spending within an ACO such as pre-existing relationships between physicians and/or prior clinical integration among the organizations forming an ACO or the available supply of specialists in the area. For example, ACOs in the two lowest quartiles of PCP focus include a substantially larger numbers of specialists per 1000 beneficiaries, a measure that may reflect stronger integration of primary and specialty care. An example of this is the Billings Clinic in Montanta, where the ACO exists within an already established, highly integrated delivery system.

These limitations notwithstanding, our findings have several implications for stakeholders. For ACO leaders, our results suggest that having PCPs provide a greater percentage of the evaluation and management services may not be a pivotal determinant of whether these organizations can achieve early cost savings. Futures studies will need to evaluate for which conditions population-level utilization and costs may be lower when specialists play a greater role providing evaluation and management services (e.g., congestive heart failure patients receiving care in cardiology clinics) [17]. There are several reasons why inclusion of a greater number of specialists may aid in reducing inpatient utilization and costs of care. First, aligning specialists with ACO priorities will likely increase communication and care coordination and reduce fragmentation of care. Second, increased engagement of specialists may place greater financial incentives on the delivery of high value care, including decreased utilization and reduced costs of care while maintaining quality. Inclusion of specialists in ACOs may also improve the breadth of services provided within an ACO, thereby limiting the need for patients to receive care outside the reach of the ACO. While this study does not provide specific answers to this question, the overall findings motivate a deeper assessment of the relative cost-efficiency of primary and specialty care in ACOs, and how this varies across specific conditions and patient populations. Such information may help to guide the distribution of PCPs and specialists within ACOs.

For policymakers, these data should encourage more detailed beneficiary-level analyses with longer follow-up that may provide greater detail and motivating factors surrounding our early findings. Understanding the structural features of an ACO that facilitate appropriate utilization and lower cost care will become increasingly important as CMS encourages renewing MSSP ACOs to move toward the two-sided risk model, while also introducing the Next Generation ACO program that involves even greater risk sharing by ACO providers [18].

## Conclusions

Moving forward, careful assessment of ACO structure and longitudinal spending patterns will inform success within the MSSP. Our findings underscore the importance of gaining a deeper understanding of the complex ways that organizational, physician, and patient characteristics influence ACO performance. Subsequent analyses will require datasets that link Medicare claims with detailed beneficiary, provider and hospital information for MSSP participants. While our study examines the policy relevant metrics of utilization and spending, we do not evaluate the cost effectiveness of the ACO model and its broader economic impact. Ultimately, such timely analyses of the comparative performance of MSSP ACOs will provide essential feedback for payers, physicians and policymakers as these organizations expand in number and assume increasing financial risk.

## Abbreviations

ACA: Affordable Care Act
ACO: Affordable Care Act
CMS: Centers for Medicare and Medicaid Services
MSSP: Medicare Shared Savings Program
PCP: Primary care physician.
